# Hypoxemia of the lower limbs during robot‐assisted radical prostatectomy in Trendelenburg position

**DOI:** 10.1002/bco2.296

**Published:** 2023-10-09

**Authors:** Lorine Haeuser, Mara Münker, Ulrich H. Frey, Marina Klaaßen, Joachim Noldus, Rein‐Jüri Palisaar

**Affiliations:** ^1^ Department of Urology and Neuro‐Urology, Marien Hospital Herne Ruhr University Bochum Herne Germany; ^2^ Department of Anesthesiology, Surgical Intensive Care, Pain and Palliative Care, Marien Hospital Herne Ruhr University Bochum Herne Germany

**Keywords:** ankle‐brachial index, compartment syndrome, hypoxemia, robot‐assisted radical prostatectomy, Trendelenburg position

## Abstract

**Objectives:**

The objective of this study is to assess frequency and risk factors for intraoperative hypoxemia of the lower limbs during robot‐assisted radical prostatectomy (RARP). Trendelenburg position during RARP may contribute to hypoxemia and compartment syndrome (CS) of the lower limbs as a major but rare complication.

**Patients and methods:**

This prospective study included patients undergoing RARP for prostate cancer. Preoperative calculation of the ankle‐brachial‐index (ABI) was performed. Peripheral oxygen saturation (SpO2) at the toes was routinely measured. Occurrence of SpO2 levels of <90% was defined as hypoxemic events and treated immediately. Blood pressure, intraabdominal pressure, SpO2 of the upper limb and surgery time were monitored in case of hypoxemia. A multivariable logistic regression model was performed with age, BMI, nicotine abuse, MAP, comorbidities as covariates and hypoxemia of the lower limbs as the outcome.

**Results:**

A total of 207 patients were included. Among these, 126 patients had ABI measurements with 10.6% having an abnormal ABI value. One, two or at least three events of lower limb hypoxemia occurred intraoperatively in 19.7%, 14.8% and 16.9%, respectively. In 20 events, surgical instruments were affecting vascular perfusion by compression. None of the covariates were statistically significant associated with lower limb hypoxemia. No patient developed a compartment syndrome.

**Conclusion:**

Decrease in oxygen saturation of the lower extremities was observed frequently during RARP, without revealing any risk factors for its occurrence. Routine oximetry leads to an early detection of hypoxemia of the lower extremities, giving the anaesthesiologist and surgeon the opportunity to make adequate adjustments (increasing blood pressure and ending iliac vessel compression).

## 1 INTRODUCTION

Acute compartment syndrome of the lower limbs following RARP in Trendelenburg position is a rare, but major, complication, with an incidence of 0.29%.^1^ While most references discussing this topic are single case studies, there exists so far only one multicentre study from the United Kingdom analysing the prevalence of risk factors in patients with lower limb compartment syndrome after RARP.^1^, ^2^, ^3^ The key event is ischemia with consecutive muscle edema in the lower extremities, resulting in increased pressure within fascias of the lower leg. In a vicious cycle, this leads to decreased blood supply and finally to necrosis and permanent loss of muscle and nerve function. In contrast to injury or trauma, the well‐leg compartment syndrome is secondary to positioning of the previously healthy legs during surgery, leading to reperfusion after a period of poor blood flow.^4^, ^5^ In order to optimize visualization and access to the pelvis during RARP, the patient is placed in 30° Trendelenburg position, which is known to decrease perfusion of the lower extremities.^6^ Matsen^7^ demonstrated that the local arteriolar pressure decreased by 0.78 mmHg for each 1 cm of ankle elevation above the right atrium. A preexisting peripheral artery disease (PAD), diagnosed via ankle‐brachial index (ABI), a non‐invasive blood pressure measurement of lower and upper extremities, might aggravate this.^8^, ^9^ The objective of this study was to assess the frequency and predictors of lower limb hypoxemia during RARP using pulse oximetry continuously in order to adjust blood pressure, avoid surgical vessel compression in case of a hypoxemic event and subsequently prevent patients from compartment syndrome.

## 2 PATIENTS AND METHODS

### 2.1 Study design

In this prospective single centre study, we aimed to expose risk factors for the occurrence of hypoxemia of the lower extremities during Trendelenburg position in patients undergoing RARP for PCa. Primary outcome was the occurrence of a hypoxemic event of the lower limbs, defined as an oxygen saturation level below 90%. This threshold value has been chosen in accordance with two Danish anaesthesiologic studies investigating incidence, duration and postoperative outcomes of intraoperative hypoxemia.^10^, ^11^ Patient demographic data included age and BMI. Medical history included the information of nicotine abuse and comorbidities according to the Charlson Comorbidity Index: myocardial infarction, congestive heart failure, peripheral vascular disease, cerebrovascular accident or transient ischemic attack, dementia, chronic obstructive pulmonary disease, mild liver disease and diabetes mellitus (1 point each); hemiplegia, chronic kidney disease, localized solid tumour, leukaemia and lymphoma (2 points each); and metastatic solid tumour and AIDS (6 points each). Procedures were performed by six urologic surgeons, using the DaVinci X or Xi System (Intuitive Surgical, Sunnyvale, CA, United States). Surgical experience was divided into three groups: low volume (<100 RARP cases), medium volume (100–500 RARP cases) and high volume (>500 RARP cases).

### 2.2 Intraoperative procedures

The patients were placed in supine position on a vacuum mattress (VAC‐PAC 8, Tap Med, Habichtswald‐Ehlen, Germany) with heels and knees padded with gel cushions. After initiation of anaesthesia with thiopental, fentanyl and rocuronium according to the standard of the Department of Anesthesiology and Surgical Intensive Care, the operating table was inclined until 30° Trendelenburg position was reached, controlled by a digital angle metre. The legs of the patients were not separated as in a lithotomy position. The positioning of the patient was the same and independent of the DaVinci system (X or Xi) used. A pulse oximeter (SpO_2_ Sensor Nellcor, Covidien, Mansfield, MA, United States) was attached to one finger and both toes to measure the arterial oxygen saturation. All vital parameters, including the systolic, diastolic and mean arterial pressure (MAP), were measured in dorsal position before and 5 min after induction of anaesthesia and in Trendelenburg position. The anaesthetic agent used was standardized. The Hasson technique was used for laparoscopic entry. The capnoperitoneum was established at a pressure of 10 mmHg and hold throughout the intervention using the PneumoSure XL insufflator (Stryker, Kalamazoo, MI, United States). In case of hypoxemia, the following parameters were recorded: SpO_2_ toe, SpO_2_ finger, side of event (right/left), systolic, diastolic, MAP and intraabdominal pressure, duration of operation and possible iatrogenic compression of the iliac vessels due to an instrument (yes/no).

### 2.3 Preoperative procedures

After 81 RARP cases, we included the ABI calculation. The day before surgery, we measured the ABI using a pocket vascular Doppler (Huntleigh Dopplex D900, Cardiff, Wales, United Kingdom). The patient was placed in dorsal position; a blood pressure cuff was inflated above the systolic pressure first at the upper arm and then at the ankle. The Doppler probe was placed above the brachial artery for the upper limb and the dorsalis pedis artery or posterior tibial artery for the lower limb. While slowly lowering the pressure in the cuff, the Doppler probe detected an acoustic signal, indicating the value of systolic blood pressure. Using this method, we measured the systolic blood pressure for each extremity and calculated the ABI using the formula: ABI = $\frac{\text{systolic blood pressure ankle}}{\text{systolic blood pressure}\;\mathrm{arm}}$. ABI readings are categorized as abnormal (≤0.90), borderline (0.91–0.99), normal (1.00–1.40) or noncompressible (>1.40).^12^

### 2.4 Postoperative procedures

Patients were seen and examined by their urologist in the week following the operative procedure and then every 3 months to be able to follow up on postoperative complications occurring after hospitalization.

### 2.5 Statistical analyses

To test for normality, we used the Shapiro–Wilk test. Mean and standard deviation were calculated to describe normally distributed continuous variables, median and interquartile range (IQR) for non‐normally distributed continuous variables, and frequencies and proportions for categorical variables. A multiple logistic regression analysis with low peripheral oxygen saturation (SpO_2_ < 90%) as outcome variable was performed, accounting for all patient characteristics, ABI, console time and surgeon's experience. All analyses were performed using Stata IC 16 (StataCorp, College Station, TX, United States), testing hypotheses with two‐sided *t* tests for continuous normally distributed variables, Mann–Whitney U test for continuous non‐normally distributed variables and Pearson's chi‐squared test for categorical variables, with alpha set at 0.05.

## 3 RESULTS

### 3.1 Patient characteristics

A total of 207 patients undergoing RARP were included in our study. Among these, 126 patients had complete ABI measurements. Twenty‐two patients (10.6%) showed an ABI < 1.0: on the left extremity (*n* = 11; 50%), right extremity (*n* = 14; 63.6%) or bilaterally (*n* = 3; 13.6%). The patients' baseline characteristics and perioperative parameters, grouped by occurrence of intraoperative hypoxemic event, are available in Tables 1 and 2, respectively. At the time point of surgery, 109 (52.7%) suffered from hypertension needing medication, and three patients (1.4%) had a known PAD. Pelvic lymph node dissection was performed in 84% of the patients. Patients who developed hypoxemia of the lower limbs during RARP more often had an abnormal ABI (11.7% vs. 9.6%) and nicotine abuse (25.8% vs. 18.4%), without statistical significance (*p* = 0.66 and *p* = 0.63, respectively).

**TABLE 1:** Patient characteristics.

	No intraoperative event (SpO_2_ ≥ 90%) *n* = 104	Intraoperative event (SpO_2_ < 90%) *n* = 103	*p* value
Age (years, median (IQR))	64 (59–70)	64 (60–68)	0.57
BMI (kg/m^2^, median (IQR))	27.2 (24.8–29.9)	27.6 (25.3–30.2)	0.30
CCI (*n*, %) 0 1 ≥2	74 (71.2) 21 (20.2) 9 (8.7)	80 (77.7) 11 (10.7) 12 (11.6)	0.14
Nicotine abuse (pack years, *n* [%]) 0 ≤20 21–40 > 40	85 (81.6) 10 (9.2) 8 (8.0) 1 (1.1)	76 (74.2) 13 (12.5) 11 (10.8) 3 (2.5)	0.63
Abnormal ABI (*n*, %)	10 (9.6)	12 (11.7)	0.66

Abbreviations: ABI, ankle brachial index; BMI, body mass index; CCI, Charlson Comorbidity Index; IQR, Interquartile range.

**TABLE 2:** Perioperative characteristics.

	No intraoperative event (SpO_2_ ≥ 90%)	Intraoperative event (SpO_2_ < 90%)	*p* value
MAP before induction of anaesthesia (mmHg, mean [SD])	100.5 (11.2)	101.2 (10.1)	0.62
MAP after induction of anaesthesia (mmHg, mean [SD])	84.5 (11.0)	84.5 (13.3)	0.96
MAP in 30° Trendelenburg position (mmHg, mean [SD])	86.4 (14.4)	84.1 (12.9)	0.24
Console time (minutes, median (IQR))	182 (57.5)	174 (50)	0.73

Abbreviations: MAP, mean arterial pressure; SD, standard deviation.

### 3.2 Perioperative parameters

In 103 of 207 patients (49.8%), at least one event of low SpO_2_ of the lower extremity was detected. Forty patients (19.7%) developed exactly one event, 30 patients (14.8%) developed two and 35 patients three or more events (16.9%), resulting in a total of 209 recorded hypoxemic events. Median console time was 178 min (IQR 54). The mean preoperative MAP in dorsal position was 100.8 mmHg (SD 10.7), after induction of anaesthesia 84.5 mmHg (SD 12.1) and in 30° Trendelenburg position 85.2 mmHg (SD 13.7). The mean intraabdominal pressure during an event was 8.5 mmHg (SD 4.3). Scrutinizing the operative duration, 25%, 50%, 75% and 100% of events occurred after 42, 75, 105 and 270 min, respectively. The left side was more often affected and then the right side with 48.3% versus 34.0%. In 9.3% of the events, both sides were affected. In 20 of the recorded 209 events of hypoxemia (9.6%), the surgeon reported a compression of the iliac vessels by an instrument as the cause of decrease in perfusion of the lower extremity. The hypoxemic events did not occur in correlation with (extended) lymph node dissection. The patient was never taken out of Trendelenburg position during hypoxemia. If an event occurred, the mean MAP was 77.6 mmHg (SD 8.9). In 75% of the events, the MAP was <83 mmHg. When analysing variables possibly influencing the outcome of blood oxygen saturation decrease below 90%, neither an abnormal ABI value nor patient characteristics, console time or surgical experience presented a statistically significant association (Table 3). We tested for collinearity for the blood pressure values with negative result. For that reason, we included all three MAP variables in the regression analysis.

**TABLE 3:** Multivariable logistic regression analyses predicting low oxygen saturation of the lower extremity for patients undergoing robot‐assisted radical prostatectomy for prostate cancer.

Variable	Blood oxygen saturation < 90% Odds Ratio (95% CI)	*p* value
ABI ≥1 <1	Ref. 1.35 (0.51–3.54)	0.54
MAP in dorsal position	1.01 (0.98–1.04)	0.54
MAP after induction of anaesthesia	1.00 (0.97–1‐02)	0.84
MAP in 30° Trendelenburg position	0.99 (0.97–1.01)	0.35
Age	0.99 (0.95–1.04)	0.73
BMI	1.05 (0.97–1.14)	0.25
CCI 0 1 ≥2	Ref. 0.53 (0.23–1.26) 1.12 (0.39–3.20)	0.15 0.83
Surgery time	1.00 (0.99–1.00)	0.33
Surgical experience High volume Medium volume Low volume	Ref. 1.68 (0.82–3.42) 1.41 (0.36–5.47)	0.15 0.62

Abbreviations: ABI, ankle brachial index; CI, confidence interval; Ref., reference.

## 4 DISCUSSION

In our study, we assessed the occurrence of intraoperative hypoxemia of the lower limbs as a precursor event of CS in RARP. While a CS is a rare complication after RARP in Trendelenburg position, occasional intraoperative hypoxemia is a common event. In our study sample of 207 patients, we could not identify risk factors from medical history or perioperative parameters.

A retrospective multicentre study of 17 institutions in the United Kingdom evaluated the incidence of lower limb compartment syndrome and prevalence of risk factors in patients undergoing RARP.^1^ They found nine cases of compartment syndrome out of 3110 patients included in the study, representing an incidence of 0.29%. Risk factors for the development of compartment syndrome were a console time over 4 h in eight of nine cases, early learning curve (3/9 cases), obesity with a BMI greater than 30 kg/m^2^ (5/9 cases), incorrect positioning of the patient (1/9 cases) and PAD (2/9 cases). In accordance to this study, several studies underscored the influence of the operative duration on the occurrence of a compartment syndrome, and surgeons should be aware of a higher risk in procedures lasting more than 4 h.^13^, ^14^ This fact is underlined by a multicentre study of the International Radical Cystectomy Consortium (IRCC) as well as two case reports describing the occurrence of compartment syndrome after robot assisted radical cystectomy, a technically challenging procedure in steep Trendelenburg position that often lasts more than 6 h. The authors discuss the type of leg holder during the intervention, long duration of surgery and preexisting PAD as main risk factors for the compartment syndrome post RC.^15^, ^16^, ^17^ In our analysis, console time was not statistically significant associated with hypoxemia of the lower limbs. Scrutinized by time point of surgery, we found 25% of events occurring during the first 42 min, 50% in 75 min, 75% in 105 min and 100% in 270 min.

In a small prospective study with 30 participants, Takechi et al.^18^ evaluated the perfusion of the lower limb via near‐infrared spectroscopy, a method to measure tissue ischemia. A change of more than eight percentage points in oxygen saturation of the lower limb was deemed clinically relevant. The group could not show any decrease in regional saturation of oxygen during RARP. The benefits of using a pulse oximetry to measure the lower limb perfusion during surgery have been discussed in the literature with contrary results. A case report by Clay and Dent^19^ revealed arterial pulses and normal oxygen saturation in two patients with compartment syndrome after limb surgery and concluded that normal pulse oximeter readings cannot exclude a compartment syndrome. On the other hand, previous works showed the importance of pulse oximetry in early detection of vascular compromise of a limb.^20^, ^21^ A prospective study of patients with limb fractures demonstrated pulse oximeters being able to confirm presence or absence of adequate blood flow distal to a fracture, determining a possible compartment syndrome.^22^ Negri et al.^20^ described the diagnosis of compartment syndrome after radial fracture with palpable pulse but loss of arterial oxygen saturation in pulse oximetry, leading to immediate fasciotomy. For this reason, diagnosing a compartment syndrome is often a combination of clinical signs (pain, motor strength and sensation) and diagnostic measurements (pulse oximetry, near‐infrared spectroscopy and intracompartmental pressure).

A limitation of our study is the small number of patients; with a total of 207 patients and only 22 patients having an abnormal ABI, it is not possible to make a valid conclusion about a potential relationship between an abnormal ABI and low blood oxygen saturation of the lower limbs in Trendelenburg position. Besides, ABI measurement is missing for the first 81 patients in our cohort. Furthermore, the degree of Trendelenburg position (24°/26°/28°/30°) might impact the occurrence of hypoxemic events and should be varied in coming studies. Another limitation might be that vital parameters were measured only in case of hypoxemic event and not routinely at standardized time points of the procedure, for example, 30 min, 1 h after capnoperitoneum, after release of capnoperitoneum and again in neutral position. Unfortunately, we did not measure the exact duration of the single hypoxemic events; however, the great majority of the events only lasted several seconds before being resolved. More studies, especially with a larger number of patients, are needed to further evaluate this possible relationship. The number of well‐leg compartment syndrome in RARP is probably underestimated with less severe cases misdiagnosed as deep vein thrombosis or neuropraxia, and the best treatment is prevention.

## 5 CONCLUSIONS

Occasional intraoperative hypoxemia is a common event during RARP for prostate cancer with up to 50% of the patients experiencing a short reduced perfusion of the lower extremities. Importantly, in our cohort, there was no association between occurrence of hypoxemia and compartment syndrome of the lower extremities. The use of pulse oximetry detects perioperative hypoxemia of the lower limbs at an early stage during RARP in the Trendelenburg position and gives both anaesthesiologists and urologists the opportunity to take countermeasures (adjust blood flow and pressure, and positioning of instruments) to avoid further complications. Nevertheless, further studies are needed investigating the influence of the positioning (lithotomy vs. supine position), the degree of Trendelenburg position and the amount of pneumoperitoneum pressure.

## CONFLICT OF INTEREST

The authors declare no conflict of interest.

## AUTHOR CONTRIBUTIONS

**Lorine Haeuser**: Conceptualization; methodology; validation; formal analysis; data curation; writing—original draft; writing—review and editing; visualization. **Mara Münker**: Methodology; data curation; writing—original draft; writing—review and editing. **Ulrich H. Frey**: Methodology; writing—original draft; writing—review and editing. **Marina Klaaßen**: Methodology; writing—review and editing. **Joachim Noldus**: Writing—review and editing; supervision. **Rein‐Jüri Palisaar**: Conceptualization; writing—review and editing; project administration.
